# Stack LSTM-Based User Identification Using Smart Shoes with Accelerometer Data

**DOI:** 10.3390/s21238129

**Published:** 2021-12-05

**Authors:** Do-Yun Kim, Seung-Hyeon Lee, Gu-Min Jeong

**Affiliations:** School of Electrical Engineering, Kookmin University, 77 Jeongnung-ro, Seongbuk-gu, Seoul 136-702, Korea; doyune525@kookmin.ac.kr (D.-Y.K.); qiaiqiai2@kookmin.ac.kr (S.-H.L.)

**Keywords:** stack LSTM, user identification, smart shoes

## Abstract

In this study, we propose a long short-term memory (LSTM)-based user identification method using accelerometer data from smart shoes. In general, for the user identification with human walking data, we require a pre-processing stage in order to divide human walking data into individual steps. Next, user identification can be made with divided step data. In these approaches, when there exist partial data that cannot complete a single step, it is difficult to apply those data to the classification. Considering these facts, in this study, we present a stack LSTM-based user identification method for smart-shoes data. Rather than using a complicated analysis method, we designed an LSTM network for user identification with accelerometer data of smart shoes. In order to learn partial data, the LSTM network was trained using walking data with random sizes and random locations. Then, the identification can be made without any additional analysis such as step division. In the experiments, user walking data with 10 m were used. The experimental results show that the average recognition rate was about 93.41%, 97.19%, and 98.26% by using walking data of 2.6, 3.9, and 5.2 s, respectively. With the experimental results, we show that the proposed method can classify users effectively.

## 1. Introduction

With the development of wearable devices, big data, and artificial intelligence, human activity recognition has been greatly developed [1,2]. Among them, human walking data include intrinsic information for human activity recognition and can be utilized in various applications such as health care, sports game analysis, human behavior analysis [3,4,5,6,7,8,9].

In order to recognize human behavior using wearable devices, many studies have been made using smart bands [10,11], smartphones [12,13,14,15,16], and smart shoes [17,18,19,20,21,22,23,24,25,26].

Wearable devices such as smart bands and smartphones can be used to recognize human behavior to a certain level of performance [10,11,12,13,14,15,16]. However, there were limitations in using human direct walking information. Hence, smart shoes are becoming essential sensors for distinguishing user behaviors [17,18,19,20,21,22,23,24,25,26]. Since smart shoes can reflect human walking information touching the ground directly, we can gather relatively useful information to analyze user behaviors. In order to classify human behaviors, we can analyze accelerometer data, pressure data, and gyroscope data from smart shoes.

Smart shoes also play an important role in identifying users using human walking data. In [22,23], user-identification methods based on smart shoes data have been presented. In [22], user identification was performed using null-space-based linear discriminant analysis(NLDA) after dividing each step based on pressure data and accelerometer data. Extending the results in [22], in [23], a deep-learning-based approach was proposed for the step detection and classification using three kinds of data (pressure data, accelerometer data, and gyroscope data). In the previous results, user identification has been performed based on the step detection. Hence, when there exists partial data that cannot complete a single step, it is difficult to apply those data to the classification.

In this study, we propose a stack LSTM-based [27,28] user identification method using accelerometer data from smart shoes. An LSTM-based identification network was designed without any complicated analysis of human gait or walking phases. With the learning of accelerometer data directly, partial data that cannot complete a single step can be used for the user identification in the proposed method. By training the designed LSTM network using accelerometer data with various window sizes and locations, the walking characteristics of users were trained to the designed LSTM network, and user identification with partial data can be performed. The experimental results were derived from the walking data of 16 users, and it was shown that the proposed method identifies users effectively.

The remainder of this article is organized as follows. In Section 2, we summarize related works regarding smart shoes and walking data analysis. In Section 3, we present the LSTM-based user identification method using accelerometer data. In Section 4, experimental results are given for the proposed method. In Section 5, experimental results are compared to the previous results. The conclusion follows in Section 6.

## 2. Related Works

### 2.1. Smart Shoes

**Figure 1:**
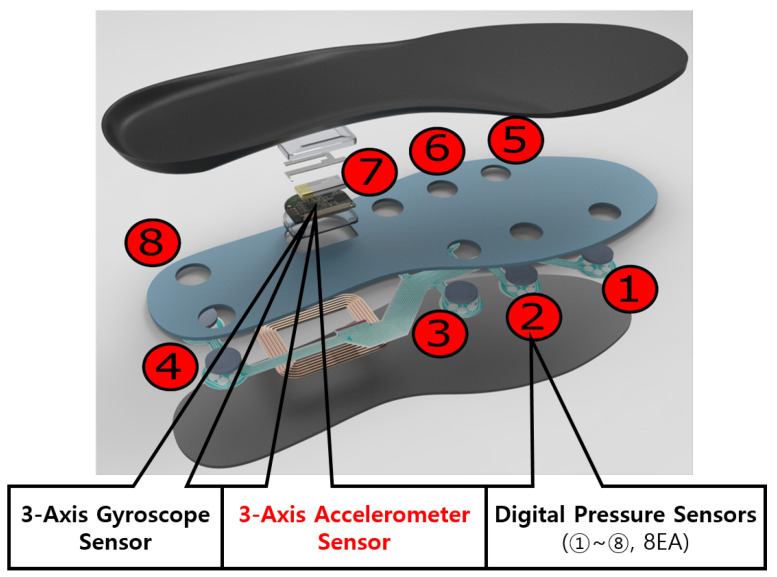
The Footlogger smart insole.

In this study, we used the “Footlogger” insole sensor module for smart shoes developed by 3L Labs Co., Ltd. (Seoul, Korea). This includes a tri-axial accelerometer, eight pressure sensors, and a gyroscope inside the insole sensor module. Using a Bluetooth connection between the smartphone and the smart shoes, we can collect data in the smartphone. Figure 1 shows the structure of the insole sensor module.

Recently, various studies using smart shoes have been conducted [17,18,19,20,21,22,23]. Three types of walking activities were classified in [17] using smart-shoes data. In [19], stride counting and walking distance estimation in human walking were performed.

**Figure 2:**
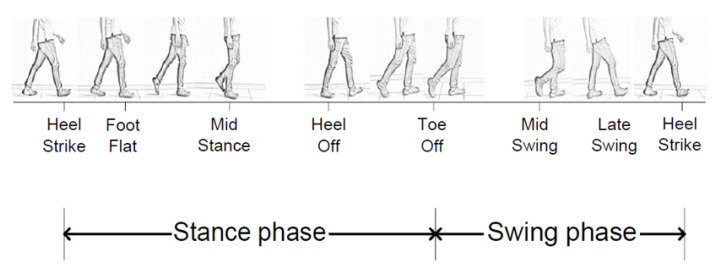
Phases of a typical gait cycle.

**Figure 3:**
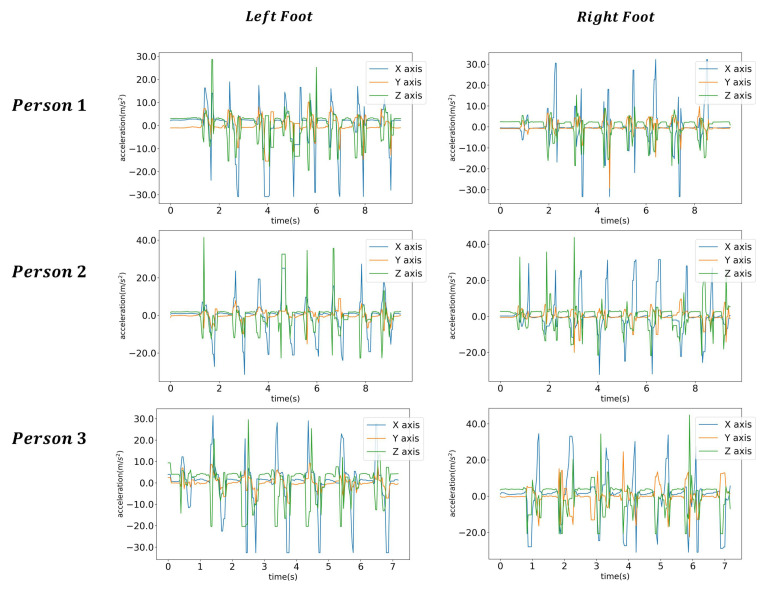
Exemplary accelerometer data for the experiment.

Generally, pressure sensors are closely related to step detection and are used for recognizing step detection [16,17,19,22,23]. Figure 2 shows the gait step cycle. In this study, we utilized only accelerometer data, and no further analysis of step detection was required. Figure 3 shows the exemplary walking data of three individuals.

### 2.2. Walking Data Analysis

As gait analysis provides essential information for human behavior recognition, there have been many studies regarding walking data analysis [17,18,19,20,21,22,23,24,25,26]. Various wearable sensors have been used for analyzing walking types [17], predicting related diseases [24], measuring walking distance [19], and estimating walking speeds [20].

Recently, there have been many research studies regarding user identification based on walking data analysis [16,22,23]. In [16], user identification was performed using accelerometer and gyroscope data obtained from smartphones. The walking period was extracted from the walking data, and each set of walking data was divided based on the walking period. The divided walking data was given as an input to the recurrent neural network (RNN) for user identification.

User-identification methods based on smart shoes data have been presented in [22,23]. In [22], pressure data and accelerometer data were used to identify users. After processing the pressure data, each step was divided. User identification was performed using the divided steps based on NLDA and a one-nearest neighborhood (NN) classifier. Extending the results of [22], in [23], three kinds of data (pressure data, accelerometer data, and gyroscope data) were used for the classification. First, classification results were extracted using a convolutional neural network (CNN) and RNN based on the walking data. Then, the final classification was performed considering the results of the CNN and RNN.

In the previous studies, step detection was required for user classification. In this article, we present a user-identification method based on LSTM utilizing accelerometer data as the input to the classification system. The entire user-identification process was conducted without step detection or gait analysis, and a simple LSTM model is presented for the classification.

### 2.3. LSTM Model

**Figure 4:**
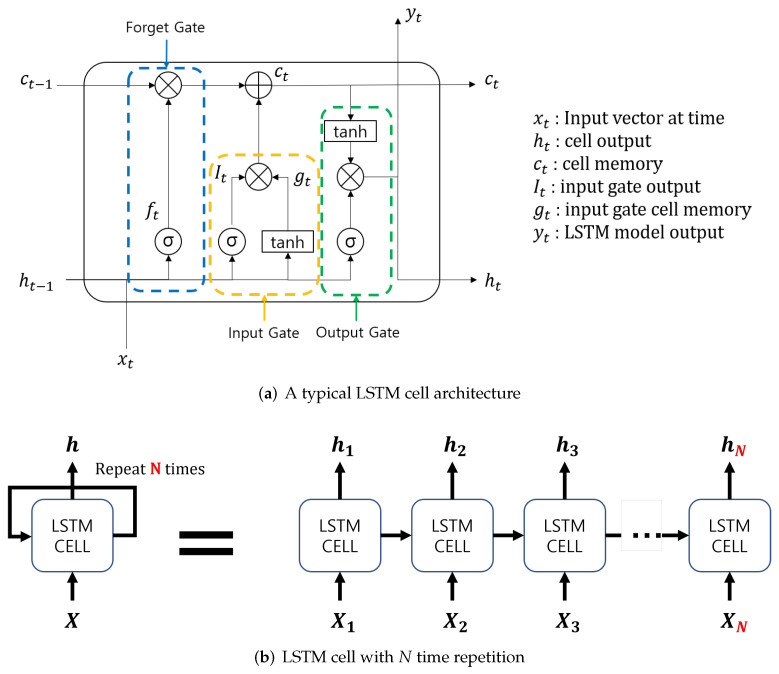
General LSTM architecture.

LSTM model is a sequential model that processes sequence data such as natural-language and time-series data overcoming the vanishing gradient problem of RNN [27]. Figure 4a shows a typical model of an LSTM cell structure. As in Figure 4b, LSTM were trained *N* times in order to have an output *h* for the input *x*. Due to these characteristics, LSTM is useful for the learning of various lengths of data.

**Figure 5:**
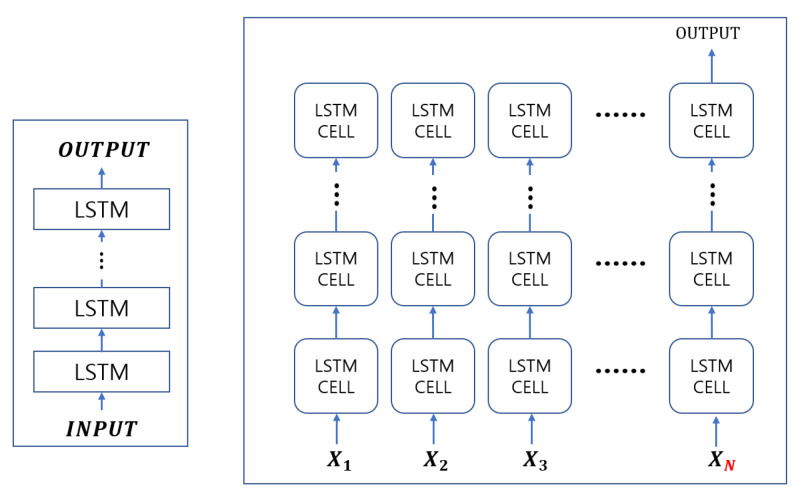
Stack LSTM architecture.

Stack LSTM is an extension of general LSTM models and files up the LSTM layer in order to process complicated models [28]. Figure 5 shows an exemplary model of a stack LSTM network.

## 3. LSTM-Based User Identification with Random Window Sizes and Random Locations

In this study, we propose an LSTM-based user identification method using accelerometer data of smart shoes. In particular, we present a classification method that can learn partial data that and does not require gait analysis before learning. Accelerometer data with random window sizes and random locations can be directly used as the input for learning and classification. Neither step detection nor gait analysis is required in the proposed method. Additionally, partial data that cannot complete a single step or more steps can be used for the classification.

Individuals have different walking speeds, stride lengths, and other unique characteristics. To allow the LSTM model to learn such characteristics without the need for preprocessing, walking data with various sizes and different locations should be given as inputs to the LSTM model.

Considering these facts, in the proposed method, we applied learning data with variable window sizes and random locations to the stack LSTM model. Through this, the stack LSTM model can learn partial data with different sizes and locations. Hence, partial data can be used for the classification without any step detection.

**Figure 6:**
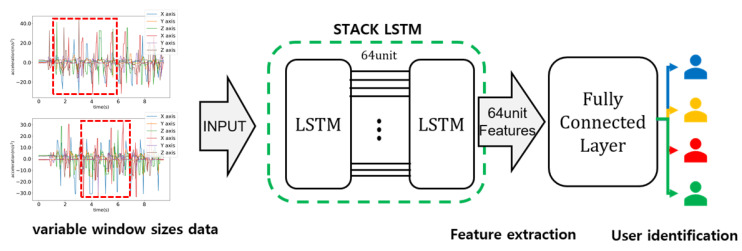
Stack LSTM network with an input of a random size and random location for the user identification.

Figure 6 shows the overall LSTM architecture of the proposed method. In the proposed method, we utilized a 2-layer stack LSTM model. Learning data with random size and location were given as an input to the stack LSTM model. A certain number of features to identify users were extracted from the stack LSTM model, and user identification was made using a fully connected layer.

### 3.1. Input Data Selection Based on Variable Window Size for the LSTM Network

**Figure 7:**
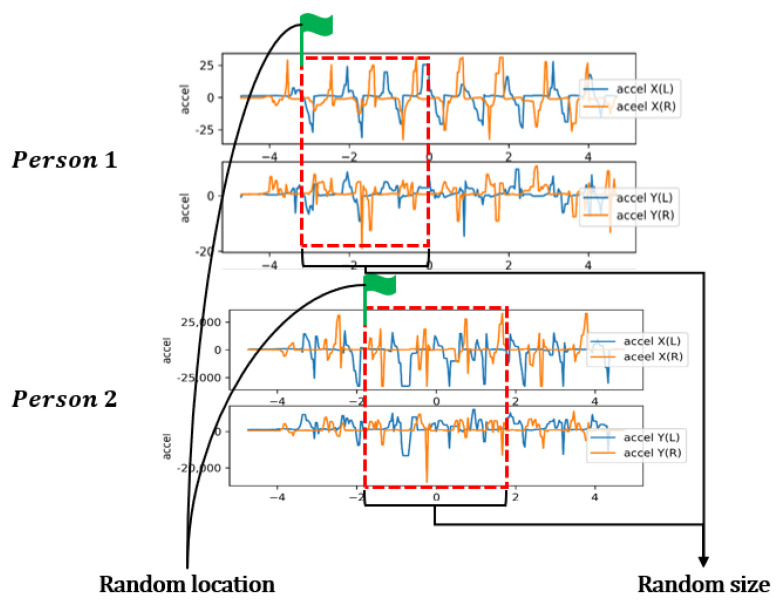
Data selection with a random location and a random size.

**Figure 8:**
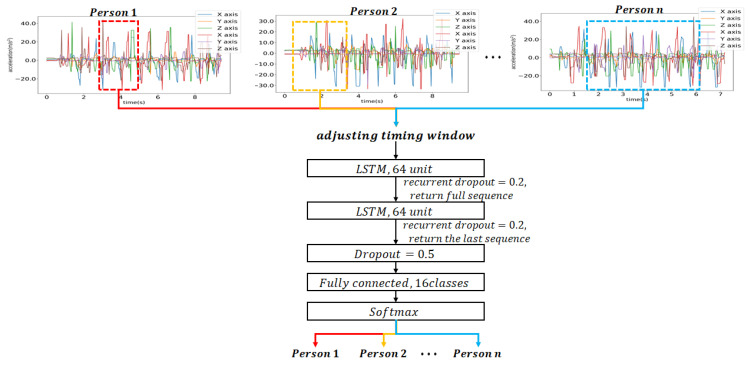
The designed LSTM network considering the variable window sizes.

In the proposed method, partial data were selected from a random location with a random size for learning, as shown in Figure 7. The selected data were utilized as inputs to the designed stack LSTM network, as shown in Figure 8. Rather than using all the data directly, randomly selected data were applied for the learning. By selecting data from a random location of random sizes, we can enable the LSTM model to learn the different walking characteristics of different users.

In the implementation of stack LSTM network in this study, we used the variable window sizes from 20 to 200. As the sampling frequency for the smart shoes was 30 Hz in this experiment, the window sizes of 20 and 200 corresponded to 0.67 s and 6.67 s, respectively. The designed LSTM network can learn the walking patterns of each user based on these settings.

### 3.2. Stack LSTM Architecture for User Identification Based on Variable Window Size

A stack LSTM network was designed in order to learn randomly selected data. Figure 8 shows the overall architecture of the designed LSTM network in our experiment. As the learning was able to proceed without any pre-processing, the overall identification architecture can be relatively simple. The designed stack LSTM network consists of two consecutive LSTM layers and one fully connected layer. From the two consecutive LSTM layers, we can extract various features for user walking patterns. One fully connected layer was used for the user classification.

In the implementation of this study, the first LSTM layer had an input shape with random sizes from 20 to 200 and provided a vector output with a size 64, which is a full sequence for the subsequent LSTM layer. The second LSTM layer produced 64 features, which was used as an input to the fully connected layer. Two-layer LSTM can enhance the learning performance compared to 1-layer LSTM. If more layers will be used, the complexity can be also increased. Hence in the proposed method, 2-layer LSTM was used.

In order to prevent overfitting, the two LSTM layers applied a recurrent dropout to 0.2, and the final LSTM output vector applied a dropout to 0.5. The fully connected layer had *n* outputs and applied softmax for the normalized probability calculation. Here, *n* is the number of users to be identified. With the input of accelerometer data, we can identify users with the proposed method.

**Figure 9:**
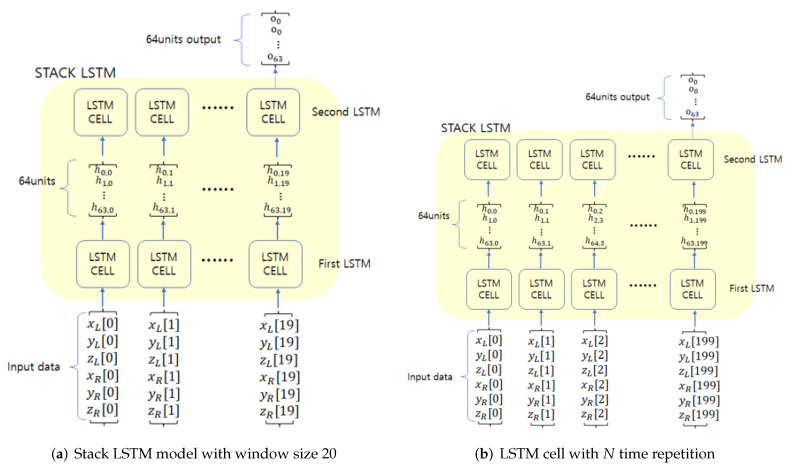
Stack LSTM model with window size 20 and 200.

Figure 9 shows the detailed architecture of the stack LSTM network used in this study. The designed LSTM network had an input data with a random size between 20 and 200. Since there were three axes for *x*, *y*, and *z* in the accelerometer, we used six accelerometer data of $x_{L}$, $y_{L}$, $z_{L}$, $x_{R}$, $y_{R}$, and $z_{R}$ for the left foot and the right foot. The stack LSTM network gave 64 features for the classification, and these features were used as an input to the fully connected layer.

## 4. Experimental Results

### 4.1. Gait Data Gathering and Preprocessing

In order to evaluate the performance of the proposed method, we applied the proposed method to walking data collected from the “Footlogger”. In this experiment, we collected only the accelerometer data with a sampling frequency of 30 Hz. Three-axis data for *x*, *y*, and *z* were considered, and $x_{L}$, $y_{L}$, $z_{L}$, $x_{R}$, $y_{R}$, and $z_{R}$ for the left foot and the right foot were collected. The accelerometer data were normalized with mean 0 and standard deviation 1.

**Figure 10:**
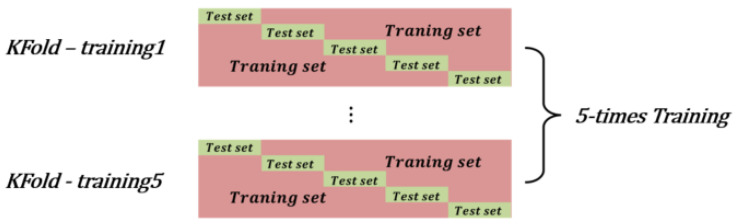
Dataset division into training set and test set using five-fold cross-validation.

We collected data from 16 people, consisting of eleven men and five women, whose ages were between 20 and 30. Each user walked 10 m 10 times at a normal walking speed. In total, 160 samples corresponding to 1600 m were collected. As the walking speeds can differ between people and the number of walking times, the data sizes can differ consequently. The data length of the fastest walking was 216 (about 7.2 s) and that of the slowest walking was 290 (about 9.6 s). As shown in Figure 10, the classification performance of the 160 data samples was assessed using a five-fold cross-validation strategy. Two samples per each user data, which are a total of 32 samples, were used as the test data, and the remaining 128 samples were used as the training data. After performing the experiment five times in five-fold cross validation, all of the data samples of each section were used as test data at least once. By shuffling the dataset randomly, we made the five-fold cross-validation experiments five times. The average value of the classification rate was calculated as the final result.

### 4.2. Performance Evaluation for User Identification

Figure 3 shows the exemplary accelerometer data used in this study. As in Figure 7, selected data from a random location of random sizes were utilized for the LSTM learning.

**Figure 11:**
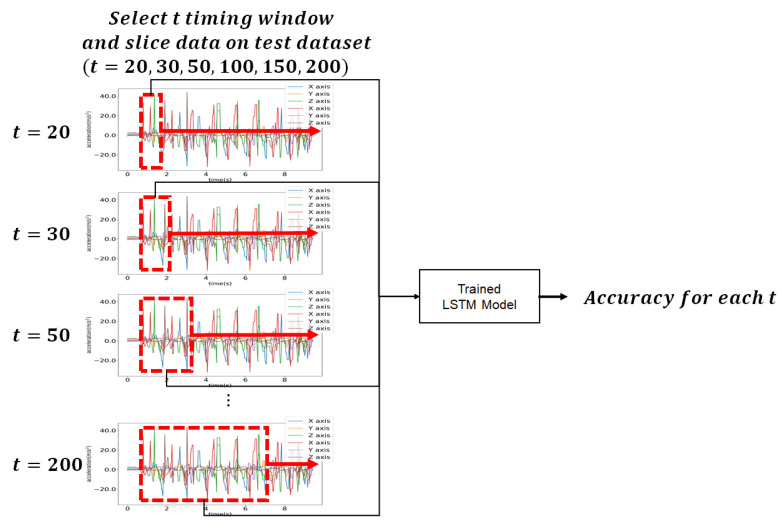
Test data selection for performance evaluation.

In this experiment, the number of epochs was 1000, and for each epoch, the number of batches was 36,828, which represents the total amount of data. The adjusting window size was between 20 and 200. Performance evaluation was made for the test data where the window sizes were 20, 30, 50, 100, 150, and 200, as shown in Figure 11. User identification was performed for each window size. Here, *T* denotes the window size of the data.

Table 1 shows the classification rate. Walking data with window sizes of 20 and 200 produced classification rates of 81.83% and 99.87%, respectively. When the window size was greater than 100, the classification rate was over 98.38%. This indicated that the classification was effectively made.

## 5. Discussion

To demonstrate the relative performance of the proposed method, we compared it with the method in [22]. The method in [22] performs step detection using pressure sensors first, and then normalizes it for the recognition. Since the proposed method in this study uses only accelerometer data and applies them for the learning without any modification, these two methods cannot be compared directly. Instead, as in Table 2, we used two, three, and four steps for the method in [22], respectively. Here, two, three, and four steps account for 26%, 39%, and 52% of the total data. Hence, for the comparison, we used input data with timing window sizes of 42, 63, and 84 accounting for 26, 39, and 52% of the total data for the proposed method. Table 2 shows the recognition rates of the two methods. The recognition rate of the proposed method was 97.19% for 39% of the total data, whereas that of the method in [22] was 92.10% for the three steps.

From the experimental results, we can see that user identification can be effectively made with the partial data from the accelerometer sensors of smart shoes. Considering the proposed method, we can derive the following advantages and disadvantages.

First, using only accelerometer data, user identification can be performed with the proposed method. Without using all sensor data of the smart shoes including accelerometer, pressure, and gyroscope sensors, the classification can be made using only accelerometer data. Additionally, partial data that cannot complete a single step or multi-steps can be used for the classification. It can be more practical considering real applications.

For the implementation, 2-layer LSTM can consume more time than the conventional machine learning based approaches. In future work, implementation in embedded systems should be considered over deep learning accelerators. Additionally, since the proposed network learns the accelerometer directly, it may not be robust to the speed variation of users. In future work, network design considering speed variation should be required.

## 6. Conclusions

In this study, we proposed a stack LSTM-based user identification method with accelerometer data of smart shoes. Through the learning of variable size and random location data, the stack LSTM model could learn partial data with different sizes and locations. Selecting data with random sizes and random locations enabled the LSTM model to learn the different characteristics of user walking patterns. Neither step detection nor gait analysis was required in the proposed method. Additionally, partial data that cannot complete a single step or multi-steps could be used for the classification. Further, the simulation results showed that the proposed method identified users effectively. Since the proposed method has a relatively simple learning architecture and is easy to implement, the proposed method can be applied to the user identification method effectively.

In this study, we assumed that the subjects walked at a normal speed. Therefore, further study of user identification at different walking speeds or in different walking environments remains needed.

## Figures and Tables

**Table 1:** User identification rate regarding timing window size.

Timing Window Size (s)	User Identification Rate (%)
20 (0.67)	81.83
30 (1.00)	89.91
50 (1.67)	95.21
100 (3.34)	98.38
150 (5.00)	98.73
200 (6.67)	99.87

**Table 2:** Recognition results of the proposed method and the method in [22].

User Identification Rate (%)			
**The Method in [22]**		**Proposed Method**	
**Number of Steps**	**Accuracy**	**Data Rate (%)**	**Accuracy**
2	82.93	26	93.41
3	92.10	39	97.19
4	93.79	52	98.26

## Data Availability

The data are not publicly available due to privacy restriction.

## References

[1] Moscato V., Picariello A., Spelí G. (2019). Community Detection based on Game Theory. EAAI.

[2] Amato F., Moscato V., Picariello A., Sperlí G. Recommendation in Social Media Networks. Proceedings of the 2017 IEEE 3rd International Conference on Multimedia Big Data.

[3] Haji Ghassemi N., Hannink J., Roth N., Gaßner H., Marxreiter F., Klucken J., Eskofier B.M. (2019). Turning Analysis during Standardized Test Using On-Shoe Wearable Sensors in Parkinson’s Disease. Sensors.

[4] Liang S., Feng D., Yikang Y., Zhe S. (2019). The Effect of Treadmill Walking on Gait and Upper Trunk through Linear and Nonlinear Analysis Methods. Sensors.

[5] Zhu C., Sheng W. Recognizing Human Daily Activity Using a Single Inertial Sensor. Proceedings of the 2010 8th World Congress on Intelligent Control and Automation.

[6] Mukhopadhyay S.C. (2015). Wearable Sensors for Human Activity Monitoring: A Review. IEEE Sens. J..

[7] Lara O.D., Labrador M.A. (2013). A Survey on Human Activity Recognition Using Wearable Sensors. IEEE Commun. Surv. Tutor..

[8] Shah S.A., Fan D., Ren A., Zhao N., Yang X., Tanoli S.A.K. (2020). Seizure Episodes Detection via Smart Medical Sensing System. J. Ambient. Intell. Humaniz. Comput..

[9] Mendes J.J.A., Vieira M.E.M., Pires M.B., Stevan S.L. (2016). Sensor Fusion and Smart Sensor in Sports and Biomedical Applications. Sensors.

[10] Chen Y.P., Yang J.Y., Liou S.N., Lee G.Y., Wang J.S. (2008). Online Classifier Construction Algorithm for Human Activity Detection Using a Tri-axial Accelerometer. Appl. Math. Comput..

[11] Nguyen N.D., Truong P.H., Jeong G.-M. (2017). Daily Wrist Activity Classification Using a Smart Band. Physiol. Meas..

[12] Győrbíró N., Fábián Á., Hományi G. (2015). An Activity Recognition System for Mobile Phones. Mob. Netw. Appl..

[13] Chen Z., Zhu Q., Soh Y.C., Zhang L. (2017). Robust Human Activity Recognition Using Smartphone Sensors via CT-PCA and Online SVM. IEEE Trans. Ind. Inform..

[14] Lee H.-H., Choi S., Lee M.-J. (2015). Step Detection Robust against the Dynamics of Smartphones. Sensors.

[15] Ho N.-H., Truong P.H., Jeong G.-M. (2016). Step-Detection and Adaptive Step-Length Estimation for Pedestrian Dead-Reckoning at Various Walking Speeds Using a Smartphone. Sensors.

[16] Fernandez-Lopez P., Liu-Jimenez J., Kiyokawa K., Wu Y., Sanchez-Reillo R. (2019). Recurrent Neural Network for Inertial Gait User Recognition in Smartphones. Sensors.

[17] Jeong G.-M., Thruong P.H., Choi S.-I. (2017). Classification of Three Types of Walking Activities Regarding Stairs Using Plantar Pressure Sensors. IEEE Sens. J..

[18] Truong P.H., You S., Ji S.-H., Jeong G.-M. (2020). Wearable System for Daily Activity Recognition Using Inertial and Pressure Sensors of a Smart Band and Smart Shoes. Int. J. Comput. Commun. Control.

[19] Truong P.H., Lee J., Kwon A.-R., Jeong G.-M. (2016). Stride Counting in Human Walking and Walking Distance Estimation Using Insole Sensors. Sensors.

[20] Feigl T., Kram S., Woller P., Siddiqui R.H., Philippsen M., Mutschler C. (2020). RNN-Aided Human Velocity Estimation from a Single IMU. Sensors.

[21] Huang B., Chen M., Huang P., Xu Y. Gait Modeling for Human Identification. Proceedings of the IEEE International Conference on Robotics and Automation.

[22] Choi S.-I., Moon J., Park H.-C., Choi S.T. (2019). User Identification from Gait Analysis Using Multi-Modal Sensors in Smart Insole. Sensors.

[23] Moon J., Minaya N.H., Le N.A., Park H.-C., Choi S.-I. (2020). Can Ensemble Deep Learning Identify People by Their Gait Using Data Collected from Multi-Modal Sensors in Their Insole?. Sensors.

[24] Pham M.H., Elshehabi M., Haertner L., Del Din S., Srulijes K., Heger T., Synofzik M., Hobert M.A., Faber G.S., Hansen C. (2017). Validation of a Step Detection Algorithm during Straight Walking and Turning in Patients with Parkinson’s Disease and Older Adults Using an Inertial Measurement Unit at the Lower Back. Front. Neurol..

[25] Zhang B., Jiang S., Wei D., Marschollek M., Zhang W. State of the Art in Gait Analysis Using Wearable Sensors for Healthcare Applications. Proceedings of the IEEE/ACIS 11th International Conference on Computer and Information Science.

[26] Gouwanda D., Senanayake S.M.N.A. Emerging Trends of Body-Mounted Sensors in Sports and Human Gait Analysis. Proceedings of the 4th Kuala Lumpur International Conference on Biomedical Engineering.

[27] Hochreiter S., Schmidhuber J. (1997). Long Short-Term Memory. Neural Comput..

[28] Dyer C., Ballesteros M., Ling W., Matthews A., Smith N.A. (2015). Transition-Based Dependency Parsing with Stack Long Short-Term Memory. ACL Anthol..

